# A Gigantic War Work

**Published:** 1918-08

**Authors:** 


### TEXAS MEDICAL JOURNAL

DR. F. E. DANIEL,
Founder.

Established July,
1885

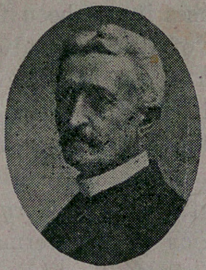

MRS. F. E. DANIEL,
Publisher and Managing
Editor.

VOL. XXXIV No. 2
AUSTIN,
AUGUST, 1918.

PUBLISHED MONTHLY.
Subscription:
$1.50

The publisher is not responsible for views of contributors.

ASSISTANT EDITORS:

DR. JOHN S. TURNER, Dallas, Texas.
DR. E. B. PARSONS, Palestine, Texas.
DR. A. P. HOWARD, Houston, Texas.
DR. JOE BECTON, Greenville, Texas.
DR. M. F. BLEDSOE, Port Arthur.
DR. J. H. FLORENCE, Dallas, Texas.
DR. J. H. REUSS, Cuero, Texas.

*"All of good the past hath had,*
*Remains to make our own time glad."*—WHITTIER.

ARMY

MEDICAL

SEP 23 1947

LIBRARY

### EDITORIAL.

### A Gigantic War Work.

The annual report of [illegible]hairman of the Committee on Medicine and Sanitation of the Council of National Defense shows an almost incredible amount of work done in the short period of the existence of the committee. Due to its activity the Medical Reserve Corps has been increased from approximately 1,800 to nearly 22,000, of which number most are now in active duty. At first it may be thought an easy matter to get a volunteer medical corps of that size, but when one stops to think of the sacrifices made by so many of the physicians and surgeons in giving up large practices and severing all family and social ties, such an array of medical talent in so short a time is little short of marvelous; for be it understood that men of best physical and professional ability have been obtained.

The work of standardization of essential medical and surgical supplies and equipment so as to increase speed and decrease cost of production is another large accomplishment of this committee. Only in this way has it been made possible to meet the enormously increased requirements of the Army and Navy.

The committee has given close study to the increase of medicinal products and especially to the use of substitutes for those products for which this country has so long depended upon Germany. In many cases it has been found that the products could be obtained here of just as good quality as those coming from Germany and in many instances substitutes have been found that take the places of German products, these in some cases proving better than the German products. Among the results obtained is the successful cultivation of digitalis of a species different from that grown in Germany, but which proved pharmaceutically superior to the German species. Another is the use of an antitoxin that will rob the gas bacillus of its horrors and will greatly aid in the surgical treatment of infected wounds. Another is the making of an ear protector of hard rubber that is inexpensive and that is already doing valuable aid on injuries to the ear in French and English hospitals. Equally important is a protector for the ear from injuries due to high explosives. In the study and application of antiseptics and disinfectants much progress has been made within the past fifteen months, and many worthless preparations have been investigated and condemned. It has become almost impossible to secure ambrine, which is extensively used in the treatment of burns, but chemists under the direction of the committee have worked out satisfactory substitutes. Satisfactory progress is being made in the production, by fermentation of starch, of acetone which is largely used in the manufacture of explosives. These are but a few of the hundreds of activities that have engaged the efforts of this committee and which show what the physicians and surgeons of the country are doing to win the war.

They have organized a nursing force available this year of nearly 100,000, and are working for a much larger reserve corps of nurses; they have made an exhaustive study of child welfare applicable equaly in times of war and peace; they have encouraged zone laws for the protection of soldiers from drink and vice; they have built up an efficient dental reserve corps; they have encouraged the medical schools to double their energies toward increasing the attendance in medical schools in order that there may continue to be an ample supply of physicians for the Army and Navy; they have organized the physicians who stay at home into a medical service corps; they have enlisted the co-operation of practically all the women physicians of the country.

All in all the report shows that the doctors of the country are responding valiantly to the call of the country and are in position to render all the necesssary medical service and research.

